# Palpitations: A Practical Approach

**DOI:** 10.7759/cureus.97748

**Published:** 2025-11-25

**Authors:** Daniel Ribero-Vargas, Juan Camilo Jaramillo-Alvarez, Jairo Gandara-Ricardo

**Affiliations:** 1 Internal Medicine, Clínica CES, Medellín, COL; 2 Internal Medicine, Universidad de Antioquia, Medellín, COL; 3 Internal Medicine, Universidad Cooperativa de Colombia, Medellín, COL; 4 Internal Medicine, Hospital Alma Máter de Antioquia, Medellín, COL; 5 Cardiology, Hospital Universitario San Vicente Fundación, Medellin, COL; 6 Cardiology, Universidad de Antioquia, Medellín, COL

**Keywords:** cardiac arrhythmia, diagnostic and therapeutic approach, electrocardiogram (ecg/ekg), electrocardiographic monitoring, heart palpitations, holter monitoring, structural heart disease (shd)

## Abstract

Palpitations are a frequent symptom in primary care, with most cases explained by benign etiologies; however, they may represent the initial manifestation of potentially fatal conditions. Psychiatric comorbidities should not be underestimated, as they may coexist with cardiac causes. The diagnostic approach is primarily clinical, requiring a systematic evaluation of history, physical examination, and a 12-lead electrocardiogram (ECG). Ambulatory ECG monitoring is indicated when clinical evaluation suggests arrhythmia or in patients with structural heart disease.

## Introduction and background

Palpitations are a frequent symptom, with most cases explained by benign entities; however, they may represent the initial manifestation of a potentially fatal condition, especially when associated with other symptoms of hemodynamic instability. [1]. The definition is challenging, but most authors describe palpitations as the unpleasant awareness of the heartbeat [2]. For some patients, the sensation is vague and sometimes difficult to specify, referred to as fluttering, skipping, or pounding in the chest or neck, either slow or rapid [3]. A complete clinical history, thorough physical examination, and systematic interpretation of the 12-lead electrocardiogram (ECG) will enable appropriate assessment of most patients, favoring organized diagnostic work and avoiding excessive additional studies [4].

As mentioned, the approach is primarily clinical. Data published by Weber et al. reveal that the use of history, physical examination, and ECG with some basic laboratory tests allowed diagnosis in 40% of evaluated patients; additionally, Holter monitoring and electrophysiological study expanded the diagnosis to 84% of patients, noting that only 43% had a cardiovascular cause for the complaint [5]. Generally, mortality associated with palpitations is low; however, 84% of patients experience recurrences during follow-up, with an unfavorable impact on quality of life [6]. Frequently, patients with palpitations are asymptomatic during medical evaluation, underscoring the paroxysmal nature of some etiologies and making diagnosis problematic [7]. Unfortunately, simultaneous ECG recording with patient-reported symptoms is unusual, which amplifies the diagnostic challenge [8]. This review will focus on palpitations of arrhythmogenic origin in the context of structural cardiac alterations.

## Review

Anamnesis and semiology of palpitations

Scientific publications have demonstrated that from the anamnesis information, the only data supporting the presence of an arrhythmogenic cause of palpitations are a history of cardiac disease (likelihood ratio (LR+) = 2.03), palpitations affecting sleep (LR+ = 2.29), or palpitations occurring during work (LR+ = 2.17); conversely, palpitation duration less than 5 minutes decreases this probability (LR- = 0.38) [9]. However, when the patient complains of a regular and rapid pounding sensation in the neck, this has an LR+ of 177 for atrioventricular (AV) nodal reentry tachycardia and an LR- of 0.07 when not reported [9]; although in other studies it was found that this could be of limited utility in differentiating nodal reentry tachycardia from other types of arrhythmia, considering that the appearance of cannon A waves is a phenomenon of AV dissociation that can occur in other arrhythmias such as ventricular tachycardia and advanced AV blocks (third degree), (LR - = 0.8) [10-12]. Despite these findings, it is useful to classify palpitations, as this can guide etiologic assessment and inform the approach [1]. It will be essential to inquire about their frequency and duration (paroxysmal or persistent nature), age of onset, mode of onset and resolution (sudden or gradual), regularity (the patient can be asked to simulate regularity by tapping a finger on a surface), associated symptoms (dyspnea, syncope), attenuating and triggering factors, personal and family history of structural heart disease, sudden death or rhythm disorders, medication and substance abuse use, and importantly, symptoms of mental illness and other systemic diseases [13]. Each of these elements is discussed below.

Duration of Palpitations

As previously mentioned, durations greater than five minutes are more suggestive of a cardiogenic origin; durations of seconds are more suggestive of premature ventricular/atrial beats, and durations of minutes are more consistent with supraventricular or ventricular arrhythmias [9].

Regularity and Frequency

These are guiding when classified into regular/irregular and rapid/slow rhythms. For example, when patients report regular and rapid rhythms, this indicates AV nodal reentry tachycardia, sinus tachycardia, or ventricular tachycardia; when rapid and arrhythmic, they suggest atrial fibrillation, atrial flutter, or atrial tachycardia with variable AV block [14]. Regular and slow rhythms favor sinus bradycardia, second-degree AV blocks, and slow and irregular rhythms could suggest atrial fibrillation with AV block and ventricular extrasystoles in any bradyarrhythmia [14].

Age of Onset

While age does not allow discrimination of palpitation etiology, the age of onset can narrow the differential diagnosis. For example, if palpitations are rapid and regular from adolescence and early adulthood, nodal or AV reentry tachycardia should be considered [15]; conversely, if they develop during old age, they are likely atrial tachycardia, atrial flutter, or atrial fibrillation [16].

Syncope

According to Albassam et al., syncope or near-syncope events are not indicative of significant arrhythmia [11]; however, in this case, the approach should focus on syncope itself rather than palpitations. It is noteworthy that in some clinical syncope scores, such as the Evaluation of Guidelines in Syncope Study [17] and as determined by the European Society of Cardiology (ESC) syncope guidelines, the presence of palpitations requires searching for their cardiogenic origin; however, this approach is beyond the scope of this review, so readers are encouraged to refer directly to the reference for further information [18]. It should be considered that when syncope is presumed to be cardiogenic in the context of palpitations, serious arrhythmias such as monomorphic and polymorphic ventricular tachycardia should be considered; however, supraventricular tachyarrhythmias can also present with this symptom, which is not always heart rate-dependent [19].

Mode of Onset and Resolution

Sudden onset and resolution of palpitations usually occur more frequently with premature beats, both atrial and ventricular, and with tachyarrhythmias such as atrial tachycardia, atrial flutter/fibrillation, and ventricular tachycardia [20]. Conversely, sinus tachycardia and palpitations associated with anxiety disorders tend to have a gradual onset and cessation [21].

Attenuating and Triggering Factors

Cessation of palpitations with Valsalva maneuvers and other vagal maneuvers suggests tachyarrhythmias dependent on the AV node, such as nodal or AV reentry; they are also commonly described during the supine position, mainly at night, and premature beats become more symptomatic, especially during left lateral decubitus, when the heart has greater contact with the thoracic wall. Therefore, the patient has greater awareness of palpitations [22]. Exercise usually suppresses premature beats but can trigger other tachyarrhythmias, such as supraventricular tachyarrhythmias, idiopathic ventricular tachycardia, and torsades de pointes in a patient with congenital long QT syndrome [23]. The relationship of symptoms with position changes such as standing up and walking, especially if associated with dizziness, syncope, or presyncope, may be related to orthostasis, which can be secondary to medications, autonomic failure associated with systemic diseases such as diabetes, amyloidosis, Parkinson-plus syndromes, hypovolemia, anemia, or postural orthostatic tachycardia syndrome when other etiologies have been ruled out [24].

Medications and Substance Abuse

Medications and substances of abuse can cause palpitations through two mechanisms: increasing adrenergic discharge or decreasing cholinergic influx, without predisposing to arrhythmias, and altering ion channel function, which may favor the appearance of both supraventricular and ventricular arrhythmias [25]. It is crucial to inquire about consumption of commonly used medications for upper respiratory infections, as many contain antihistamines and alpha agonists that can generate palpitations; additionally, it is relevant to carefully review the complete list of medications patients consume that could potentially explain the appearance of arrhythmias; the same applies to inquiring about consumption of foods and substances such as caffeine, alcohol, cocaine, amphetamines, marijuana, and energy drinks, among others [26]. Notably, it is important to inquire about medications that prolong the QT interval and thereby cause polymorphic ventricular tachycardia (torsades de pointes) [27]. Some examples are first- and second-generation antipsychotics, tricyclic antidepressants, serotonin and dual reuptake inhibitors, H1 antihistamines, prokinetics, antiemetics, antiarrhythmics, long-acting opioids with erratic absorption such as methadone, antibiotics such as azoles, macrolides, and fluoroquinolones, antimalarials, beta-2 agonists, and antineoplastics, among others [28].

While QT prolongation has been established as the alert for ventricular arrhythmia and sudden death risk, other markers have been proposed in recent years to stratify this outcome risk, such as the Tpeak-Tend interval, which is the distance between the highest point of the T wave and the point where the same wave ends; and the ratio of Tpeak-Tend to QT has shown prediction of ventricular arrhythmia risk in other pathologies such as Brugada syndrome, hypertrophic cardiomyopathy, ischemic heart disease, and congenital QT syndrome, among others [29]. A clear cutoff point has not yet been established; usually, Tpeak-Tend over QT is less influenced by heart rate, and its normal value is more consistent at 0.17-0.23 [30]. A meta-analysis found that in patients with acquired long QT in the context of medication use or bradyarrhythmias, Tpeak-Tend was significantly greater in those who had ventricular arrhythmias or sudden death compared to those who did not develop the event (165 vs. 96 milliseconds, respectively), as was Tpeak-Tend over QT (0.29 vs. 0.19, respectively) [31]. Although a precise tool to predict this risk is not yet available, it is suggested to consider these variables; hence, the importance of reviewing the ECG when patients take QT-prolonging medications and withdrawing or continuing them according to the risk-benefit ratio, indication, and the possibility of other therapeutic alternatives. It is also noteworthy that drugs causing orthostasis can also generate palpitations, as can abrupt discontinuation of beta-blockers, for which gradual dose reduction is recommended [32].

Personal and Family History

A history of structural heart disease in both family and patient should alert to a possible cardiac origin of palpitations [1]. This includes cardiomyopathies, ischemic heart disease, valvular disease, and conduction disorders in general. It is also important to inquire about sudden death history, as it is known that while this aspect does not always indicate a congenital arrhythmogenic disorder, when it involves one family member, it increases the relative risk to 1.8 for sudden death. When two family members are involved, it is 9.4 [29]. It is crucial to ask about at least three previous generations, as many channelopathies or cardiomyopathies are autosomal recessive, dominant, or X-linked. It can only be elucidated with a complete history [30]. Other significant past medical histories include conduction disorders, such as Wolff-Parkinson-White syndrome, and infiltrative diseases, such as sarcoidosis, amyloidosis, and hemochromatosis, which can cause AV and intraventricular conduction disturbances and, consequently, bradyarrhythmias and palpitations [33]. The presence of chronic obstructive pulmonary disease is associated with multifocal atrial tachycardia, not to mention the presence of atrial fibrillation and ventricular arrhythmias in this patient group [34].

It is also relevant to consider comorbidities such as anxiety spectrum disorders and depression, which are frequent in both the general population and in those patients with a history of heart disease [35]. Psychiatric disorders can trigger sinus tachycardia on their own or distort perception of heartbeats. Still, they can also lead to heightened alertness in the patient and their sensations, thereby increasing the likelihood of perceiving palpitations of cardiac or arrhythmogenic origin [36]. In turn, chronic adrenergic stimulation can predispose to the development of supraventricular tachyarrhythmias [37]. It has been shown that up to 31% of palpitation cases are attributed to a psychiatric cause; however, it has also been documented that up to two-thirds of these patients ultimately are diagnosed with a cardiac arrhythmia that is most often managed with catheter ablation [37]. It is known that the prevalence of anxiety and depression disorders in patients with heart failure can be as high as 13% and 19%, respectively, making it clear that psychiatric symptoms can favor or coexist with other causes of palpitations and should not be underestimated [35].

Classification

According to the European Heart Rhythm Association, palpitations can be classified into four groups [1].

Extrasystolic

Refers to ectopic beats that generally produce a sensation of a suddenly skipped or missed beat, with alternating periods of normal heart rhythm sensation. Particularly when of ventricular origin, the post-extrasystolic beat produces a vigorous contraction that strikes against the chest and is reported by the patient [1].

Tachycardic

These have a sudden onset and cessation, described by the patient as a fluttering sensation in the chest, accompanied by a clear increase in heart rate, and can be irregular or regular depending on the type of tachycardia. In sinus tachycardia, onset and cessation are usually more gradual. They may be accompanied by other cardiovascular symptoms such as syncope, dyspnea, weakness, and chest pain [1].

Anxiety-Related

In this type of palpitation, the onset is gradual, the heart rate is never above what is possible for age, and it is usually preceded by other symptoms such as facial paresthesias, hyperventilation, atypical chest pain, agitation, and globus sensation, among others. However, it should always be ruled out that symptoms are due to a cardiac arrhythmia, as previously emphasized [5].

Pounding

Perceived as intense and regular beats and are not particularly at elevated frequencies, although they tend to be persistent [1]. Usually related to underlying structural heart disease, such as aortic regurgitation, hypertrophic cardiomyopathy, or high cardiac output states, such as infections, hypovolemia, pregnancy, anemic heart, beriberi due to thiamine deficiency, Paget's disease, or thyrotoxicosis of any etiology, among others [4].

Etiology

Palpitations may be caused by both supraventricular and ventricular cardiac arrhythmias, AV blocks or cardiac device dysfunction, structural heart diseases such as ventricular hypertrophy or valvular heart disease, physiological states such as hyperthyroidism, anemia, or orthostatic hypotension; psychiatric disorders such as anxiety, panic, or depression; anticholinergic and vasodilator medications; or psychoactive substances, including alcohol and alcohol withdrawal, amphetamines, cocaine, and marijuana, among others [1].

Physical examination

Physical examination has limited yield in finding the origin of palpitations due to their intermittent nature, but it can provide valuable clues, especially when the etiology is systemic [38]. A thin, tachycardic, diaphoretic, warm, tremulous patient with exophthalmos or goiter suggests hyperthyroidism; pallor of mucous membranes and splenomegaly are found in anemias; pulmonary auscultation abnormalities and desaturation on pulse oximetry should suggest multifocal atrial tachycardia or atrial flutter induced by pulmonary disease or in the context of pulmonary embolism [4]. If the patient has palpitations with position changes, an orthostatic test will be mandatory [24]. An irregular pulse suggests atrial fibrillation or premature ventricular contractions with a positive predictive value of 91% for finding a significant arrhythmia on 24-hour ECG monitoring [7]. Point of maximal impulse displaced to the left suggests structural cardiomyopathy [39]. Finding a murmur requires ruling out valvulopathy, as mitral valve prolapse with a telesystolic murmur or aortic insufficiency with a decrescendo diastolic murmur are associated with palpitations [40].

Diagnostic tests

Among the diagnostic aids available in the approach to the patient with palpitations, some laboratory tests should be ordered according to clinical suspicion, highlighting diseases such as anemia, electrolyte disorders, and thyrotoxicosis, so if the clinical picture suggests it but not routinely, it will be appropriate to request a complete blood count, electrolyte panel, and TSH, respectively [4]. In the context of suspected arrhythmic disease, cardiac monitoring tools will help make the diagnosis [41]. Monitoring devices can be divided into external and implantable. External devices include the conventional 12-lead ECG, Holter monitor, hospital or ambulatory telemetry, and external event monitors. Implantable tools include the implantable event monitor, pacemakers, and implantable cardioverter-defibrillators [41].

The most important diagnostic test in the approach to a patient with active palpitations is the 12-lead ECG, as it is the reference standard for determining whether the cause of palpitations is arrhythmogenic [8]. It can also provide additional information when the patient is not actively experiencing palpitations, as it may reveal signs of relevant arrhythmogenic or structural heart diseases, like short or long cQT, epsilon waves (arrhythmogenic right ventricular cardiomyopathy), Q waves (ischemic or hypertrophic cardiomyopathy), short PR and delta waves (Wolff-Parkinson-White syndrome), among others [1]. However, most patients do not have symptoms at the time of consultation; therefore, in most cases, the ECG will be normal even if the patient has a cardiac etiology [8].

Ambulatory ECG monitoring

The International Society of Holter and Noninvasive Electrocardiology and the Heart Rhythm Society, in their 2017 consensus, propose indications for ordering ambulatory ECG monitoring (including both external and implantable devices) for palpitations [41]. This consensus establishes the following indications: when clinical history, physical examination, and ECG suggest the possibility of arrhythmias in the context of structural heart disease, family history of sudden death, or presence of a channelopathy with known arrhythmia risk; when the patient requires certainty and a specific explanation of the origin of their symptom; when symptoms require specific management and treatment (antiarrhythmics, ablation, among others); and when after a complete history and physical examination there is no explanation for the recurring symptom [41].

As mentioned, the particular device to select in this context depends on each specific case. The clinical situation in which each device is indicated will be addressed, highlighting its advantages and disadvantages.

Holter Test

It is a widely available tool; however, although it was designed to evaluate patients with palpitations, this study may not be the best choice, as its performance depends mainly on symptom frequency and pre-test probability [42]. This tool can establish a relationship between patient symptoms and causal rhythm or conduction disorders, provided the patient is diligent in symptom diary reporting; however, in those who do not experience symptoms at the time of testing, arrhythmias found may not necessarily be associated with palpitations [42]. It has been shown to have acceptable specificity in distinguishing arrhythmia-associated palpitations; however, its sensitivity is poor, around 10-15%, which is why other devices are available for this purpose [41]. With clear indications for requesting ambulatory ECG monitoring, the Holter monitor performs best diagnostically when the patient experiences daily or nearly daily palpitations, so it is not appropriate when palpitations are infrequent or sporadic [41]. It has some advantages, such as the ability to detect asymptomatic arrhythmias requiring intervention (e.g., atrial fibrillation), clinician familiarity with the study, and availability; however, this does not necessarily make it more cost-effective [42]. Some disadvantages include poor patient adherence to symptom diary recording for clinical-ECG correlation, electrodes can easily disconnect, artifacts can be generated by poor electrode adhesion to skin, it does not provide real-time information, monitoring is restricted to 24-48 hours, and it also limits patient routine activities that may even trigger symptoms, such as physical activity [42].

Event Monitor

These devices are primarily indicated when symptoms occur infrequently (weekly or monthly) [43]. Monitoring begins immediately when the patient attaches the device to the precordial skin and starts recording when symptoms are perceived, with the duration pre-programmed in the equipment (seconds to minutes) [43]. It has the disadvantage that if the palpitation is very short or causes patient collapse, the arrhythmia may not be recorded during the episode, as the patient will not be able to attach the device [43]. However, it has better performance than Holter monitoring, achieving sensitivities up to 50-60%, and in general terms, it is the device with the best cost-effectiveness [41].

Loop Monitor (External and Implantable) - Ambulatory Telemetric Monitoring

In this case, the device continuously monitors heart rhythm but does not continuously store the tracing information [44]. Once the patient perceives the symptom, recording is initiated. It remains attached to the patient via electrodes throughout the day and stores data for seconds to minutes before and after recording initiation. It can be activated manually by the patient and can also be configured to activate automatically if it detects an abnormal rhythm, which increases its diagnostic performance [44]. The externally used version of the device can last up to one month, while the implantable version can last up to three years, making it ideal for patients with very sporadic or short symptoms. As such, it is the ambulatory cardiac monitoring device with the highest sensitivity, but it is much more expensive and carries an added risk of local complications at the insertion site in the implantable type [41]. When a portable receiver is added to the loop device and sends ECG recordings via a mobile phone to a website or central operations, it is called ambulatory telemetric cardiac monitoring. It has the advantage of monitoring the patient's heart rhythm in real time [45].

Pacemaker and Implantable Cardioverter-Defibrillator

These are not indicated in patients with palpitations, but if the patient already has these devices implanted and presents palpitations, the device memory can be read to search for arrhythmias that could explain the patient's symptoms [41].

Electrophysiological Study

It is indicated if no diagnosis is reached with the previously mentioned tools and data suggesting significant disease (e.g., persistent palpitations affecting quality of life, an irregular pulse on physical examination, abnormal ECG, structural heart disease on echocardiogram, among others) [46]. It can also be the initial study over ambulatory ECG monitoring if the patient has baseline significant heart disease or if palpitations precede syncope [46].

Echocardiogram

This study should be requested when the clinical picture, physical examination, and ECG suggest structural heart disease, including cardiomyopathies, suspected congenital heart disease presenting in adulthood, suspected valvulopathy, evidence of ischemic heart disease, and left ventricular hypertrophy, among others [39].

Other Studies

Myocardial stress tests, either physiological or pharmacological (treadmill exercise test, exercise echocardiography, pharmacological stress echocardiography, myocardial perfusion, among others), are indicated in the clinical context of suspected coronary disease (when there is concomitant chest pain) and in patients where palpitations appear clearly related to exercise [1]. Criteria for selection will depend on the specific characteristics of each patient, a topic beyond the scope of this review.

Most patients with palpitations are in an outpatient context [1]. Studies with inpatient cardiac monitoring can be considered in patients with high suspicion or confirmation of cardiac electrical disease (Brugada syndrome) or structural disease (mitral valve prolapse), severe systemic disease (thyroid storm), need for electrophysiological study, or evidence of arrhythmia requiring invasive therapy (complete AV block, ventricular tachycardia) [1].

Diagnostic value of tests

As mentioned, the diagnostic performance of ambulatory monitoring tests depends on the specific clinical situation of each patient; in general, the literature is consistent that longer monitoring time results in higher study sensitivity [8]. Table 1 shows the performance of each study.

**Table 1:** Diagnostic performance of ECG monitoring devices Adapted from [41].

Device	Recording duration	Diagnostic yield
Holter monitor	24-48 hours	10-15%
Event monitor	60 seconds	50-60%
External loop device - ambulatory cardiac telemetry	1-4 weeks	70-85%
Implantable loop device	≤36 months	80-90%

Approach

**Figure 1:**
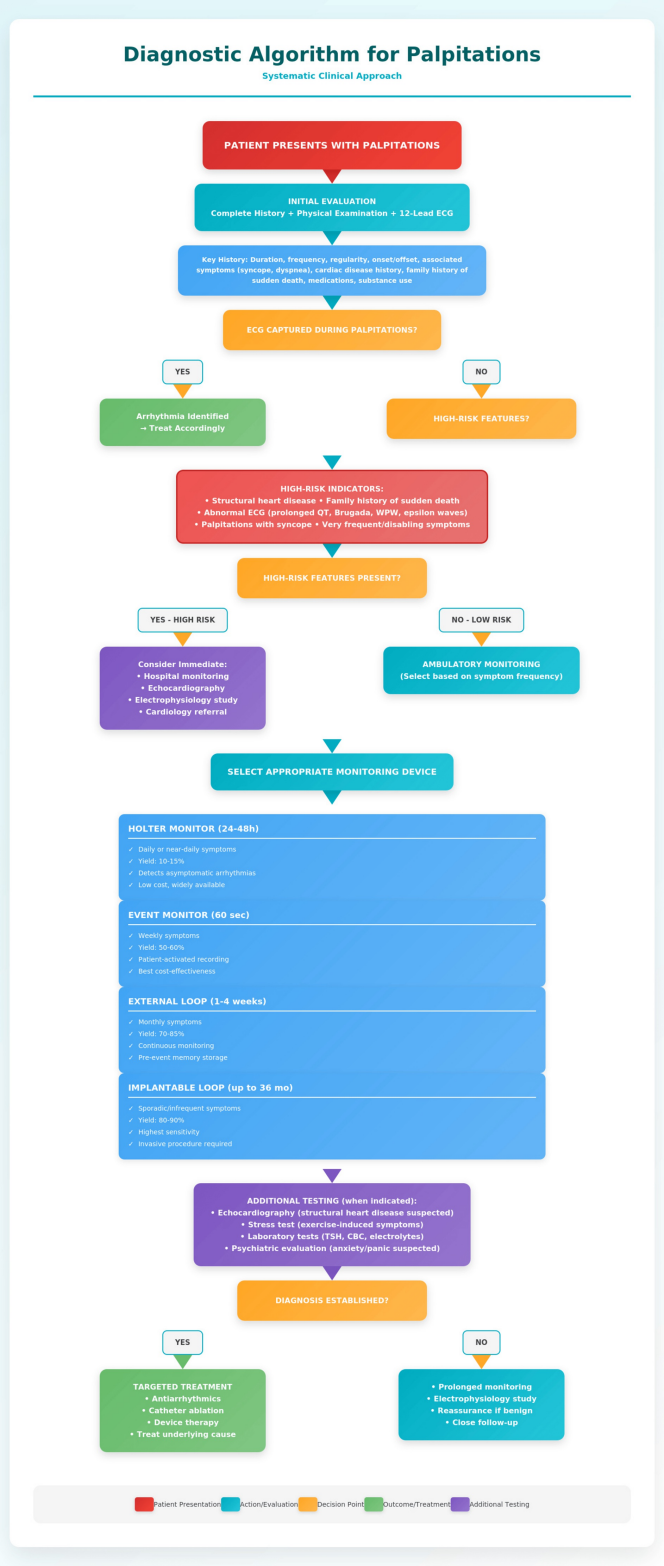
Comprehensive approach of palpitations Image Credit: Ribero-Vargas D (own elaboration). Information taken from [1].

The approach to palpitations will then depend on all the elements already mentioned, where clinical judgment dictates the path to follow. The fundamental point is to identify which patients will require more advanced studies, primarily ambulatory ECG monitoring, based on suspected significant heart disease or symptom intensity (Figure 1).

## Conclusions

Palpitations are a frequent symptom in primary care outpatient consultations. Psychiatric disorders can explain palpitations but can also favor or perpetuate an underlying arrhythmic cause and should not be underestimated. Every patient with palpitations should have a complete history and physical examination, along with a 12-lead ECG. Patients with symptoms or signs of heart disease or those without suspected heart disease but with severe and frequent symptoms should be studied with ambulatory cardiac monitoring. The ambulatory cardiac monitoring device to choose will depend on the frequency and duration of palpitations, as well as availability.
